# Intracerebroventricular Catalase Reduces Hepatic Insulin Sensitivity and Increases Responses to Hypoglycemia in Rats

**DOI:** 10.1210/en.2015-2054

**Published:** 2016-10-14

**Authors:** S. Pauliina Markkula, David Lyons, Chen-Yu Yueh, Christine Riches, Paul Hurst, Barbara Fielding, Lora K. Heisler, Mark L. Evans

**Affiliations:** Wellcome Trust/Medical Research Council Institute of Metabolic Science and Department of Medicine (S.P.M., C.-Y.Y., C.R., P.H., M.L.E.), University of Cambridge, Cambridge CB20QQ, United Kingdom; Rowett Institute of Nutrition and Health (D.L., L.K.H.), University of Aberdeen, Aberdeen AB25 2ZD, United Kingdom; Department of Family Medicine (C.-Y.Y.), Chang Gung Memorial Hospital, Chiayi, Taiwan; Chang Gung University of Science and Technology (C.-Y.Y.), Taoyuan City 33303, Taiwan; Oxford Centre for Diabetes, Endocrinology and Metabolism (B.F.), University of Oxford, Oxford OX37JT, United Kingdom; and Department of Nutritional Sciences (B.F.), University of Surrey, Guildford GU27XH, United Kingdom

## Abstract

Specialized metabolic sensors in the hypothalamus regulate blood glucose levels by influencing hepatic glucose output and hypoglycemic counterregulatory responses. Hypothalamic reactive oxygen species (ROS) may act as a metabolic signal-mediating responses to changes in glucose, other substrates and hormones. The role of ROS in the brain's control of glucose homeostasis remains unclear. We hypothesized that hydrogen peroxide (H_2_O_2_), a relatively stable form of ROS, acts as a sensor of neuronal glucose consumption and availability and that lowering brain H_2_O_2_ with the enzyme catalase would lead to systemic responses increasing blood glucose. During hyperinsulinemic euglycemic clamps in rats, intracerebroventricular catalase infusion resulted in increased hepatic glucose output, which was associated with reduced neuronal activity in the arcuate nucleus of the hypothalamus. Electrophysiological recordings revealed a subset of arcuate nucleus neurons expressing proopiomelanocortin that were inhibited by catalase and excited by H_2_O_2_. During hypoglycemic clamps, intracerebroventricular catalase increased glucagon and epinephrine responses to hypoglycemia, consistent with perceived lower glucose levels. Our data suggest that H_2_O_2_ represents an important metabolic cue, which, through tuning the electrical activity of key neuronal populations such as proopiomelanocortin neurons, may have a role in the brain's influence of glucose homeostasis and energy balance.

Glucose is a key cellular fuel essential for life whose blood concentration in healthy individuals is actively maintained within a narrow range. Hyperglycemia leads to a compensatory increase in peripheral glucose uptake and/or a decrease in hepatic glucose production (1). Hypoglycemia results in a reversal of these effects and, if blood glucose falls low enough, the triggering of an array of counterregulatory neurohumoral responses (2). The hypothalamus has a key role in regulating these homeostatic, blood glucose-controlling, mechanisms, acting via both humoral outflows (eg, increased ACTH release stimulating glucocorticoid responses to hypoglycemia) but also via autonomic neural outflow to liver, pancreas, and adrenal medulla (3). Accordingly, subsets of neurons within the hypothalamus including proopiomelanocortin (POMC) and others such as agouti-related peptide-expressing populations have been shown to respond to changes in extracellular glucose concentration (4–6) through a process likely to involve uptake and metabolism of glucose molecules (7–9). Circulating hormones such as insulin may also act in the hypothalamus to alter hepatic glucose fluxes (10), possibly acting on glucose-sensing neurons that therefore act as broader metabolic sensors.

Recent data suggest that hypothalamic reactive oxygen species (ROS) may act as a metabolic signal in response to changes in substrates and hormonal signals leading to changes in peripheral metabolism and energy balance. A rise in glucose or lipid metabolism may result in a burst of hypothalamic ROS mediating a rise in pancreatic insulin secretion and/or decreased appetite (11). Insulin may also stimulate hypothalamic ROS (12) and ROS have been implicated in peripheral insulin signaling (13). Furthermore, exogenous delivery of ROS to the hypothalamus has been shown to stimulate POMC neurons (14), cells typically activated by nutrient signals, including glucose (15). These studies have largely examined the sensing of oxidative stress in general, a process that involves a number of different molecular signals. Of the numerous types of ROS, hydrogen peroxide (H_2_O_2_) has been proposed as the most suitable candidate for a role as a messenger molecule due to its enzymatic production, degradation and limited oxidation targets (16)

In this work, we assessed the influence of hypothalamic H_2_O_2_ signaling on peripheral insulin sensitivity during euglycemia, on the triggering of hypoglycemic counterregulatory responses and the cellular activity of arcuate nucleus of the hypothalamus (ARC) POMC neurons.

## Materials and Methods

All procedures were approved in advance by both local and national ethical review processes and conducted in accordance with the United Kingdom Home Office welfare guidelines under the animal scientific procedures act (1986). Male Sprague Dawley rats (Charles River Laboratories) weighing 250–350 g were used for in vivo studies. For ex vivo electrophysiological studies, transgenic mice expressing a red fluorescent protein driven by Pomc neuronal regulatory elements (POMC^DsRed^) aged between 2 and 6 months were used (17). Standard chow and water were available ad libitum except when specified. Chemicals were from Sigma-Aldrich unless stated.

### Surgery

Vascular catheters were placed in carotid artery and jugular vein and a guide cannula was inserted stereotaxically aimed at the base of the third ventricle, 0.9 mm lateral and 2.2 mm caudal to the bregma, 8.4 mm ventral from the dura, at a 5° angle towards midline, as previously described (18). Surgery was performed under inhaled anesthesia with routine perioperative antibiotic and analgesia. Experiments were performed after full recovery, 7–10 days after surgery.

### Euglycemic clamps with ICV catalase

**Figure 1.**
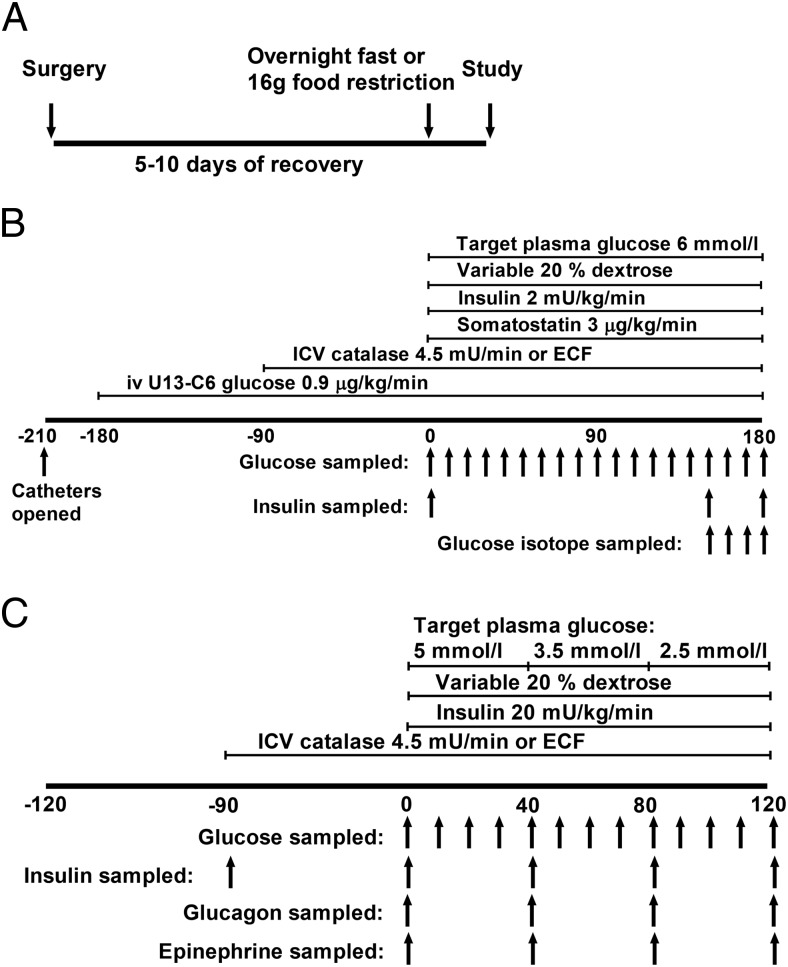
In vivo studies design. A, Rats underwent surgery for insertion of vascular catheters to carotid artery and jugular vein and a third ventricle ICV cannula. Insulin clamp studies were conducted 5–10 days later. For the night preceding the study day, the rats were restricted to 16 g of chow (euglycemic clamps) or fasted (hypoglycemic clamps). On the study day, rats were randomized to receive either ICV infusion of catalase or ECF and euglycemic (B) or hypoglycemic (C) clamps were performed as shown.

After an overnight food restriction of 16 g, catheterized rats underwent a 180-minute euglycemic “pancreatic” clamp (Figure 1, A and B). In short, a (nonprimed) continuous 2-mU/kg · min insulin intravascular infusion was delivered together with 3-μg/kg · min somatostatin and 20% dextrose, with the rate of the latter adjusted according to serial plasma glucose measurements. Additionally, rats received a 0.9-μg/kg · min infusion of carbon 13-labeled glucose tracer ([U-^13^C_6_]D-glucose; GK Gas Products Ltd) starting 180 minutes before the start of the insulin infusion (primed for first 8 minutes at 4.5 μg/kg · min) and continuing throughout the clamp. Rats also received an ICV infusion of either 4.5-mU/min catalase or control artificial extracellular fluid (ECF) starting 90 minutes before and continuing throughout the clamp. Typically, 2 animals were studied in parallel (1 control and 1 ICV catalase). During clamp studies, plasma samples were collected for analysis of insulin levels at 150 and 180 minutes and for ^13^C_6_ D-glucose at 150, 160, 170, and 180 minutes.

### Hyperinsulinemic hypoglycemic clamps with ICV catalase

After an overnight fast, catheterized rats underwent a 120-minute stepwise hypoglycemic clamp with a primed continuous 20-mU/kg · min insulin infusion together with a simultaneous infusion of 20% dextrose, with the rate of the latter adjusted according to serial plasma glucose measurements (Figure 1, A and C). The target plasma glucose was 5mM for the first 40 minutes of the clamp, 3.5mM for the second stage, and 2.5mM in the final 40 minutes. Blood sampling for assessment of insulin, epinephrine, and glucagon was done at the end of each stage. As above, the rats received either ICV infusion of 4.5-mU/min catalase or ECF starting 90 minutes before and continuing throughout the clamp, again with ICV catalase and control animals studied in parallel.

### Glucose and hormone assays

Blood glucose was measured on 5- to 10-μL plasma samples using a bench top glucose analyzer utilizing the glucose oxidase method (Analox GM9; Analox Instruments). Plasma [U-^13^C_6_]D-glucose concentration enrichments were determined using gas chromatography-mass spectrometry (19). Plasma hormone concentrations (glucagon and epinephrine) were determined using ELISA (Linco).

### Tissue collection

Forty-five to 60 minutes after completion of euglycemic clamps, the rats were euthanized with a 1-mL ip injection of sodium pentobarbital. Samples of liver were collected on dry ice for analysis of gluconeogenic enzyme expression. The brains were perfused with 10% formaldehyde, saturated with 20% sucrose and stored in PBS azide until histological confirmation of ICV cannula placement and c-Fos immunohistochemical staining as previously described (20). Mean c-Fos activation in the ARC was quantified in bregma levels −1.9 to −3.8 mm and expressed as average counts/view.

### Ex vivo electrophysiology

For electrophysiological experiments, POMC^DS-RED^ mice were anesthetized with sodium pentobarbital (Euthatal) and decapitated. The brain was rapidly removed and placed in cold, oxygenated (95%O_2_/5%CO_2_) “slicing” solution containing 214mM sucrose, 2.0mM KCl, 1.2mM NaH_2_PO_4_, 26mM NaHCO_3_, 4mM MgSO_4_, 0.1mM CaCl_2_, and 10mM D-glucose. The brain was glued to a vibrating microtome (Campden Instruments), and 200-μm-thick coronal sections of the hypothalamus containing the ARC were prepared. Slices were immediately transferred to a “recording” solution containing 127mM NaCl, 2.0mM KCl, 1.2mM NaH_2_PO_4_, 26mM NaHCO_3_, 1.3mM MgCl_2_, 2.4mM CaCl_2_, and 5mM D-glucose, in a continuously oxygenated holding chamber at 35°C for a period of 25 minutes. Subsequently, slices were allowed to recover in recording solution at room temperature for a minimum of 1 hour before recording. For whole-cell recordings, slices were transferred to a submerged chamber and a Slicescope upright microscope (Scientifica) was used for infrared-differential interference contrast and fluorescence visualization of cells. During recording, unless otherwise described, slices were continuously perfused at a rate of approximately 2 mL/min with oxygenated recording solution (as above) maintained at 32°C with an inline heater. All pharmacological compounds were bath applied. No synaptic blockers were added. Neurons whose membrane potential changed by greater than 4 mV within 10 minutes of compound application were considered responsive.

Whole-cell current-clamp recordings were performed with pipettes (3–7 MΩ when filled with intracellular solution) made from borosilicate glass capillaries (World Precision Instruments) pulled on a P-97 Flaming/Brown micropipette puller (Sutter). The intracellular recording solution contained 140mM K-gluconate, 10mM KCl, 10mM HEPES, 1mM EGTA, and 2mM Na_2_ATP (pH 7.3; with KOH). Recordings were performed using a Multiclamp 700B amplifier and pClamp10 software (Molecular Devices). Access resistance was monitored throughout the experiments, and neurons in which the series resistance was more than 25 MΩ or changed more than 15% were excluded from the statistics. Liquid junction potential was 16.4 mV and not compensated. The recorded signal was sampled at 10 kHz and filtered at 2 kHz unless otherwise stated.

Data were examined using either parametric (Student's *t* test or two-way ANOVA) or nonparametric (unpaired Mann-Whitney) tests in SPSS 23.0 with *P* < .05 as significance level. Data are presented as mean ± SEM.

## Results

### ICV infusion of catalase increases hepatic glucose output during euglycemic clamp

**Figure 2.**
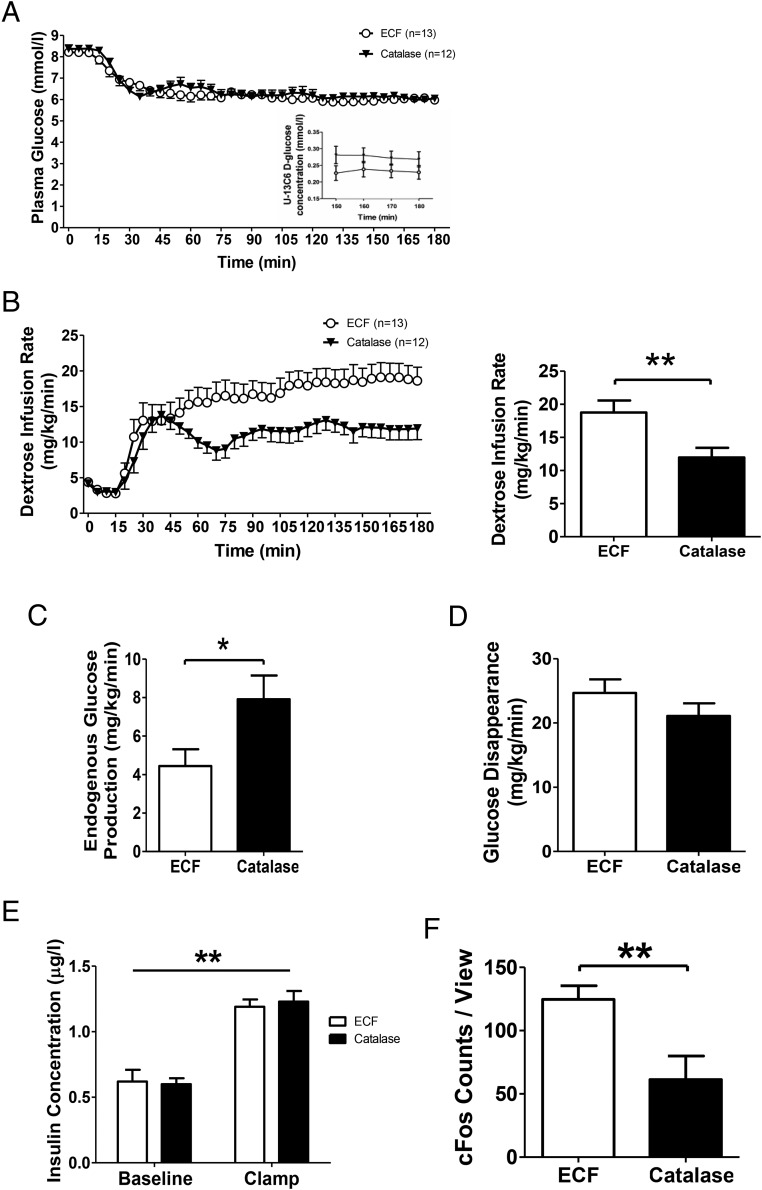
ICV infusion of catalase increases hepatic glucose output. A, Plasma glucose levels were matched between catalase and ECF groups. Inset, Plasma U-13C D-glucose levels were steady during last 45 minutes of clamps and lower in ECF group (*P* < .01; two-way ANOVA for group effect). B, Dextrose infusion rates during last 45 minutes of clamps were lower in catalase group (*P* < .001; two-way ANOVA for both time and group effects). C, ICV catalase significantly increased the steady state endogenous glucose production. D, The glucose disposal rate did not differ significantly between the 2 groups. E, Plasma insulin concentrations were similar in ECF and catalase groups throughout. F, ICV catalase reduced neuronal activation in the ARC indicated by c-Fos immunohistochemistry after hyperinsulinemic euglycemic clamp (n = 4) ICV catalase in comparison with ECF (n = 3) controls; *, *P* < .05; **, *P* < .01.

Catalase is an enzyme that breaks down H_2_O_2_, one of the most stable forms of biological ROS (16). We examined the effect of third ventricle ICV catalase infusion on glucose homeostasis during euglycemic clamps in rats. In keeping with our hypothesis of brain H_2_O_2_ acting as a sensor of glucose metabolism, ICV catalase significantly reduced the dextrose infusion required to maintain a similar level of euglycemia during the steady state of the euglycemic clamp in comparison with vehicle controls (Figure 2, A and B).

Analysis of glucose fluxes using [U-^13^C_6_]D-glucose revealed an increase in hepatic glucose output of catalase-infused rats (Figure 2C) but no difference in glucose disappearance between the 2 groups (Figure 2D).

### ICV infusion of catalase reduces c-Fos activation in the ARC

Neuronal activity in the ARC triggered by intravascular glucose has been proposed to be conditional on the production of ROS (21). After observation that ICV catalase infusion leads to systemic responses consistent with the reduced perception of glucose, we assessed the effects of ICV catalase on neuronal activation in the ARC during euglycemic clamps. We observed that third ventricle infusion of catalase significantly reduced c-Fos immunoreactivity in the ARC (Figure 2G). Although we did not examine the immunohistochemical identity of the population(s) of neurons affected, anorexigenic POMC neurons are a likely candidate as they have been shown to be activated by an increase in both glucose and its metabolic biproduct H_2_O_2_ (14, 15).

### Arcuate POMC neurons are inhibited by catalase and activated by H_2_O_2_

**Figure 3.**
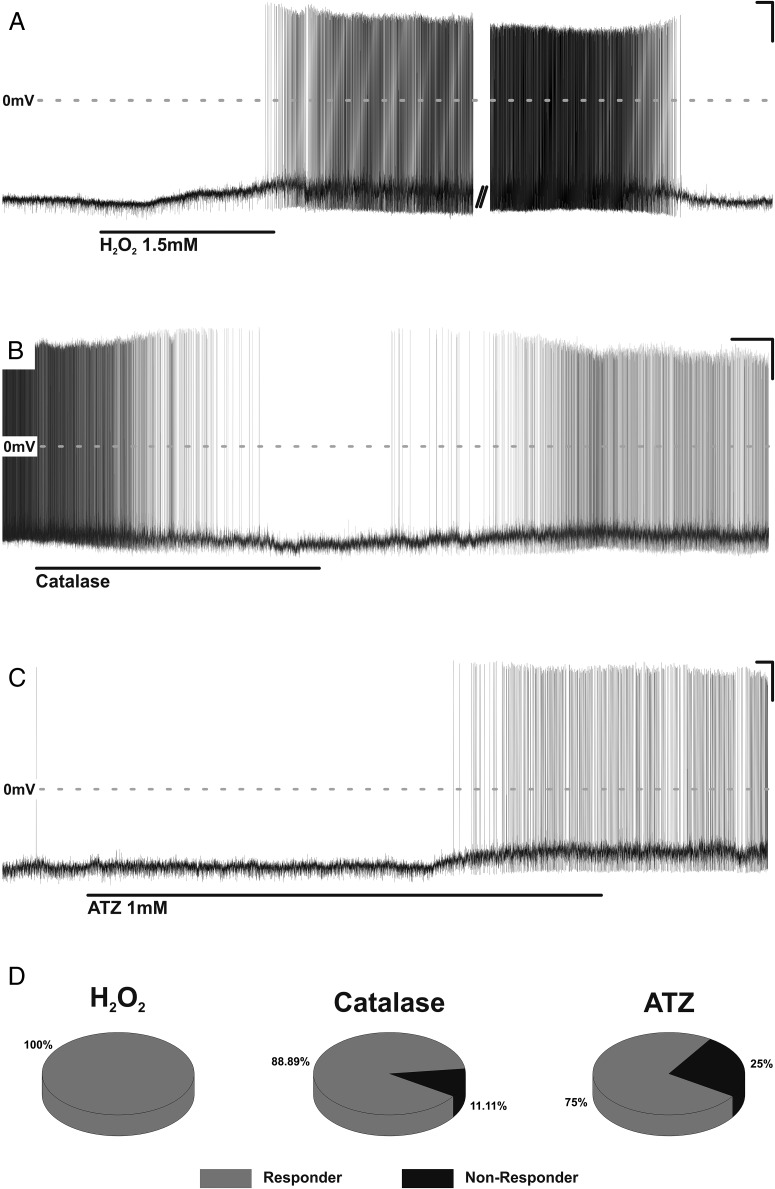
Arcuate POMC respond to H_2_O_2_, catalase and catalase inhibition. Before drug application in studies illustrated in A and C, neurons were held below threshold with negative DC current of constant amplitude (−5pA and [minus 8pA, respectively). A, Current clamp recording of an arcuate POMC neuron. Bath application of H_2_O_2_ resulted in depolarization and commencement of action potential discharge (20 mV/20 s). B, Current clamp recording of an arcuate POMC neuron. Bath application of the H_2_O_2_ scavenging enzyme catalase resulted in hyperpolarization and cessation of action potential discharge (20 mV/2 min). C, Current clamp recording of an arcuate POMC neuron. Bath application of the catalase inhibitor ATZ resulted in depolarization and commencement of action potential discharge (20 mV/20 s). D, Summary of the responsiveness of arcuate POMC neurons to H_2_O_2_ (8/8), catalase (8/9), and ATZ (3/4).

To determine the effect of H_2_O_2_ and H_2_O_2_ scavenging upon arcuate POMC neuronal activity ex vivo, we prepared acute hypothalamic brain slices from POMC^DS-Red^ animals and obtained whole-cell recordings from fluorescently identified POMC neurons. Application of 1.5mM H_2_O_2_ resulted in 100% (8/8) of neurons tested undergoing membrane depolarization and an increase in action potential discharge (Figure 3, A and D). Application of catalase (500 U/mL) induced the opposite effect, causing 89% (8/9) of neurons tested to undergo membrane hyperpolarization and an associated inhibition of action potential discharge (Figure 3, B and D). Furthermore, administration of the catalase inhibitor 3-amino-1,2,4-triazole (ATZ), which would presumably result in reduction in ROS scavenging and an associated increase in endogenous H_2_O_2_, mimicked the effect of direct H_2_O_2_ application causing a decrease in membrane potential and increase in action potential discharge in 75% (3/4) of neurons tested (Figure 3, C and D). These results demonstrate the ability of catalase availability to bidirectionally modulate the activity of a key population of glucose sensing neurons implicated in the regulation peripheral glucose homeostasis.

### ICV infusion of catalase increases counterregulatory responses to hypoglycemia

**Figure 4.**
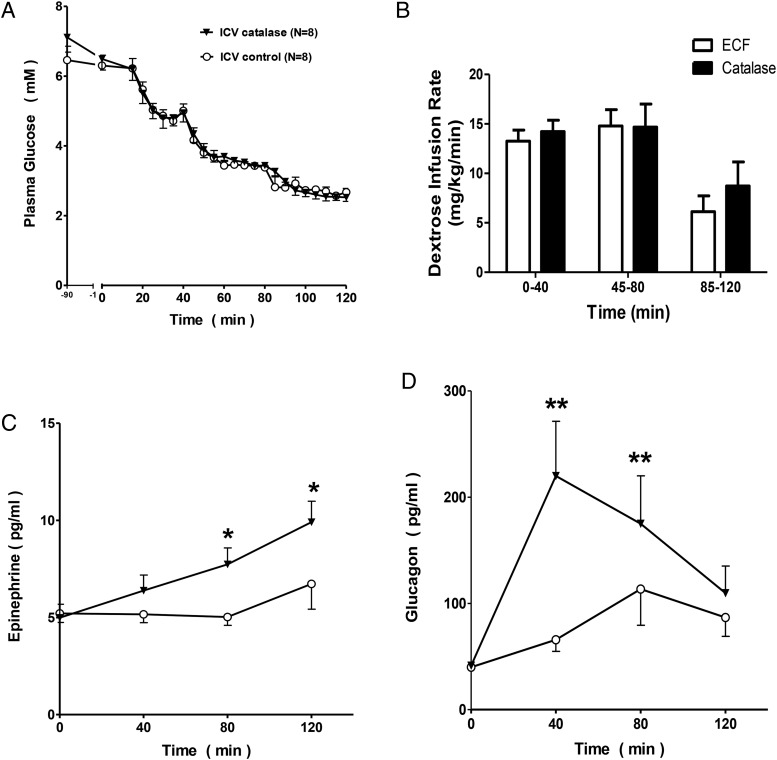
ICV infusion of catalase increased counterregulatory responses to hypoglycemia. A, Plasma glucose levels were matched between the catalase (n = 8) and ECF (n = 8) groups. B, Dextrose infusion rates did not differ between the groups for the first 80 minutes of the clamp but during the final 40 minutes, catalase-infused animals required significantly more dextrose to attain the target plasma glucose. C, ICV infusion of catalase significantly increased the epinephrine response to hypoglycemia at plasma glucose levels of 3.5mM and 2.5mM. D, The glucagon response to hypoglycemia was amplified and peaked at a higher plasma glucose level in the group receiving ICV catalase; *, *P* < .05; **, *P* < .01.

To examine the contribution of H_2_O_2_ signaling to the control of counterregulatory responses to hypoglycemia, we performed hyperinsulinemic hypoglycemic clamps in rats receiving ICV catalase. In keeping with our hypothesis that reduced H_2_O_2_ levels in the brain would be interpreted as reduced blood glucose levels, ICV infusion of catalase significantly increased the adrenaline produced as part of the counterregulatory response to hypoglycemia at plasma glucose levels of 3.5mM and 2.5mM (Figure 4C). Similarly, glucagon responses to hypoglycemia were amplified and peaked at a higher plasma glucose level in the group receiving ICV catalase, consistent with a shifted threshold for counterregulatory responses in this group (Figure 4D). In these hypoglycemic studies, the dextrose infusion rates required to maintain target plasma glucose levels did not differ significantly between the 2 groups (Figure 4B).

## Discussion

Maintaining blood glucose levels within a relatively narrow range is prerequisite for avoiding potentially fatal hypoglycemia and longer-term complications associated with hyperglycemia as seen in patients with diabetes. The brain, in particular the hypothalamus, is involved in the control of peripheral glucose homeostasis through regulating hepatic glucose output, pancreatic insulin secretion, and counterregulatory responses to hypoglycemia (10, 22, 23). Here, we show that brain H_2_O_2_ signaling is involved in the central control of glucose homeostasis through regulating hepatic glucose output and counterregulatory responses to hypoglycemia.

Our findings are in keeping with our hypothesis that a reduction in brain, and in particular hypothalamic H_2_O_2_ levels would trigger increased hepatic glucose production because of the brain perceiving glucose levels to be lower than they actually were. This is broadly consistent with data from Leloup et al (11), who proposed that a rise in blood glucose is registered through a rise in hypothalamic ROS, leading to a rise in pancreatic insulin release. If ROS are involved in brain insulin signaling (12, 13), it is also possible that the effects that we observed with ICV catalase were mediated by a reduction in the suggested central actions of insulin to restrain hepatic glucose output (10). Given the apparent overlap between glucose/substrate and insulin sensing, it is possible that brain ROS may act as a “signal of plenty” mediating both glucose and insulin signaling.

We also observed that ICV catalase delivery promoted stronger and earlier counterregulatory responses during step-wise hypoglycemic clamps. This, too, is consistent with the hypothesis that brain glucose sensing neurons use ROS as a glucose signal so that a fall in H_2_O_2_ signifies a fall in glucose. In this work, we have not identified whether effects were mediated by basomedial hypothalamic glucose-excited or glucose-inhibited neurons, or indeed both. It is likely that other brain areas outside the hypothalamus also contribute to the integration of counterregulatory responses to hypoglycemia, and it is possible that our effects may also have been mediated through these nonhypothalamic areas (24). However, given that the catalase was infused to the base of the third ventricle and given the changes in brain activation we observed, we think that it is more likely that the effects under these study conditions arose from reduced ROS levels in the hypothalamus.

Although we do not unambiguously identify the chemical phenotype of the catalase inhibited neurons, it seems likely that at least a proportion express POMC as previous work from Diano et al have shown that H_2_O_2_ acutely activates POMC neurons in the basomedial hypothalamus (14). This is supported by our ex vivo experiments which also showed that POMC neurons were excited by ROS. Moreover, we were able to show that POMC neurons were inhibited by catalase and excited by catalase inhibition.

A network of glucose sensors within and outside brain probably provide integrated information to allow the body to maintain glucose homeostasis. Within brain, a number of different glucose sensing cells have been identified, including POMC-expressing neurons. Although a key role for POMC in energy balance is clearly established, growing evidence suggests that POMC also play a role in glucose homeostasis, acting on hepatic glucose balance, insulin secretion and perhaps renal glucose excretion (25–27). Alternatively effects might be mediated by other non-POMC neurons exerting effects on energy homeostasis such as agouti-related peptide or indeed other metabolically active populations of cells in the basomedial hypothalamus. The intracellular pathways influenced by H_2_O_2_ signaling remain to be identified. Previous studies of ROS have suggested this may work through protein kinase C and the activation of ATP-sensitive potassium channels (implicated in pancreatic and brain glucose sensing (28, 29).

Of note, brain ROS signaling has also been implicated in another area of central metabolic homeostasis, acting in the brain control of osmoregulation and blood pressure. Analogous to the suggested mechanisms of action for glucose homeostasis, brain ROS have been suggested both to allow local sensing of metabolic changes (osmotic potential) (30) and the mediation of the central effects of a hormonal signal, with hypothalamic superoxide ion facilitating the actions of angiotensin II (31).

Furthermore, brain and peripheral ROS may also mediate glucose-homeostasis via actions on islet function. Normalization of brain redox status has also been shown to reverse pathologically increased glucose-induced insulin responses in obese Zucker rats (32). These findings parallel the role of ROS in induced insulin release at the level of the pancreatic β-cell (33, 34), a process with similarities to glucose sensing in a subset of hypothalamic neurons (8). Recently, ROS have also been implicated in glucose detection by the pancreatic α-cell (35).

In conclusion, our data support the growing evidence for ROS and enzymes responsible for their metabolism in the control of glucose homeostasis. Further work is needed to elucidate the molecular signaling pathways involved, to examine whether and how these processes may become altered in diabetes and whether this may even offer future therapeutic potential.
